# Health status and use of medication and their association with migration related exposures among Syrian refugees in Lebanon and Norway: a cross-sectional study

**DOI:** 10.1186/s12889-020-8376-7

**Published:** 2020-03-17

**Authors:** Elisabeth Marie Strømme, Jasmin Haj-Younes, Wegdan Hasha, Lars T. Fadnes, Bernadette Kumar, Jannicke Igland, Esperanza Diaz

**Affiliations:** 1 Department of Global Public Health and Primary Care, University of Bergen, Kalfarveien 31, 5018 Bergen, Norway; 2 Unit for Migration and Health, Norwegian Institute of Public Health, Oslo, Norway

**Keywords:** Refugees, Transients and migrants, Health status

## Abstract

**Background:**

The health of forcibly displaced individuals changes along their migration path and estimates of disease burden are essential to develop health care policies and practices adequately corresponding to their health care needs. This study aims to describe the health status and use of medication among Syrian refugees in two different migration phases: in a transit setting and in a recipient country. Further, we aim to investigate the associations between migration related exposures and both chronic pain and mental health among Syrian refugees.

**Methods:**

This is a cross-sectional study based on survey data collected among 827 adult Syrian refugees in Lebanon and Norway during 2017–2018. The survey instrument included items measuring somatic status (including chronic pain), mental health (using the HSCL-10 and HTQ items), use of medication and migration related exposures. We used descriptive statistics to calculate standardised prevalence proportions and regression analyses to study associations between migration related exposures and health outcomes.

**Results:**

The response rate was 85%. The mean age in the sample was 33 years and 41% were women. Half of the participants reported that they had never had any health problems. The prevalence of non-communicable diseases was 12%. Headache and musculoskeletal complaints were the most prevalent conditions reported, with 30% reporting chronic pain lasting for more than six months. Symptoms indicating anxiety and/or depression were presented by 35%, while 7% revealed symptoms compatible with post-traumatic stress disorder. Among those reporting non-communicable diseases a substantial share did not seem to receive adequate treatment. Trauma experiences were associated with both chronic pain and anxiety/depression symptoms, and the latter were also associated with migrating without family members.

**Conclusions:**

Migrant-friendly public health policies and practises should acknowledge migration related risks, address discontinuity in care of chronic conditions and target common complaints such as chronic pain and mental health problems among forcibly displaced individuals.

## Background

Refugees are susceptible to potential adverse health effects resulting from their vulnerable life situation. The range of health related risks facing people on the move includes stressors such as unfamiliar surroundings, uncertain prospects and factors related to financial constraint like poor nutrition and sanitary conditions [1, 2]. Furthermore, the health care services offered along their migration trajectories are often arbitrary, fragmented and difficult to access [3].

By the end of 2017, an unprecedented figure of 68.5 million individuals were forcibly displaced worldwide [4]. Some refugees have a fairly swift migration journey before arrival at their resettlement destination. However, others are on the move for a long time or stay in temporary settings, often in neighbouring countries, for years. Most of the refugees entering Europe in the last years have fled from the atrocities of the civil war in Syria. An estimated 1 million Syrian refugees registered by the United Nations High Commissioner for Refugees (UNHCR) are living in neighbouring Lebanon [4]. Although Lebanon has not ratified the 1951 Refugee Convention, it hosts the largest per capita refugee population in the world, where one in four people is a refugee [4]. Since the beginning of the conflict approximately one million Syrians have moved to Europe as asylum seekers or refugees [5] and at the beginning of 2018 about 26.000 Syrians lived in Norway [6].

The public health situation of pre-war Syria was characterized by an increasing burden of non-communicable diseases (NCDs) such as obesity, diabetes and coronary heart disease, and decreasing levels of infectious disease [7]. However, the devastation of war may have extensive impact on the epidemiological profile of a population [7]. Forcibly displaced individuals frequently experience profound psychological distress, which may manifest as mental disorders but also as somatic complaints such as pain [8, 9]. Data on the health of displaced Syrians who reside in transit situations are scarce and only a few scientific publications report on the health conditions of the refugees dwelling in the countries bordering Syria [10–13]. These studies suggest a high load of mental disorders and disruptions in the continuity of care for chronic disease, but do not offer more comprehensive epidemiological profiles of the Syrian refugees. To our knowledge, there are no estimates of disease burden among newly arrived refugees and asylum seekers in a Norwegian context. Overall, the morbidity patterns and use of medication among newly arrived forcibly displaced immigrants in Europe remain largely unexplored.

To ensure evidence-based policies and practises, transit and receiving countries need better comprehension of the health complaints and diseases affecting non-settled and newly resettled forcibly displaced individuals. Identifying key factors associated with adverse health outcomes among refugees can further aid the development of preventive measures, and enhance clinical evaluations, diagnostic procedures and treatment interventions corresponding to their needs. In this article we aim to 1) describe the health status and use of medication among two groups of Syrian refugees; one in transit in Lebanon and one shortly after arrival in Norway and 2) study the associations between migration related factors and both mental health and chronic pain among Syrian refugees.

## Methods

This cross-sectional study is based on survey data collected during 2017–2018. We recruited Syrian refugees aged 16 and above under protection by the UNHCR in Lebanon, awaiting resettlement in Norway. We also recruited early post-migration phase refugees and asylum seekers who had arrived in Norway by various routes; either as resettlement refugees, as asylum seekers on the EU-relocation scheme [14] or as asylum seekers passing through Europe or European Russia on often clandestine, perilous journeys.

The terms *refugee* and *asylum seeker* will be used interchangeably in this article as nearly all Syrian asylum applicants to Norway were granted residency during 2017–2018 [15].

In Lebanon, all adult Syrians attending mandatory educational activities offered by the International Organization for Migration were invited to participate. In Norway we approached Syrian refugees in connection with mandatory educational activities at one asylum centre, where all adults present were invited to participate, and at two educational centres for newly arrived immigrants, where we invited a sample including participants from all educational levels.

A questionnaire in Arabic was self-completed on-site. Trained staff aided those with low literacy level and Kurmanji translators assisted Syrian Kurds with limited Arabic skills. Medical staff was available to respond to any sign of re-traumatization during assessment. Participants in Lebanon received a small monetary recompense.

The overall response rate was 85% (827 of 972 invited); 93% (506 of 544 invited) in Lebanon and 75% (321 of 428 invited) in Norway. The most frequent reasons for non-participation when stated were illness, childcare, lack of interest or fear of influence on legal status.

The project was approved by the Regional Committee for Medical & Health Research Ethics of South East Norway, reference 2017/377. All enrolled participants received information about the study and signed a consent form in Arabic.

### Survey instrument

The survey included questions on somatic complaints, mental health and use of medication as well as sociodemographic and migration related factors. Questions on general health status and use of medication were obtained from The Nord-Trøndelag Health Study (HUNT) [16] and the Oslo Health Study (HUBRO) [17]. The questions from the HUNT study inquire whether respondents suffer or have suffered from a range of health conditions including NCDs and chronic impairments that have lasted more than 1 year and impede daily life. Our NCD variable combines cardiovascular diseases, chronic respiratory diseases, diabetes and cancer. The questions from HUBRO map the extent and frequency of use of various drugs. Chronic pain was assessed by a validated question from the International Association for the Study of Pain, which defines pain lasting for more than 6 months as chronic [18]. Mental health was assessed by the validated instruments Hopkins Symptom Checklist (HSCL-10) [19, 20] and the Harvard Trauma Questionnaire (HTQ) [21, 22]. These instruments have satisfactory psychometric properties in Arabic-speaking populations and have regularly been employed in surveys among refugees [22–24]. HSCL-10 rates the extent to which symptoms of anxiety and depression have distressed the respondent during the last week on a 4-point Likert scale. We used mean item score 1.85 as threshold for psychological distress (range 1–4), predicting a clinically relevant anxiety or depression [20]. HTQ rates the burden of post-traumatic stress symptoms using the same time frame and response scale as HSCL-10. We used mean item score 2.5 as cut-off for post-traumatic stress disorder (PTSD) (range 1–4) [21].

The Single General Trauma Item was used to measure exposure to traumatic events relating to the experience of forced migration [25]. To identify other exposures related to the migration process, our research team developed seven items to map various aspects of the respondent’s migration history: time since flight from country of origin, time since arrival in current country, stay in transit country (ies), time in transit country (ies), solo-migration, detainment during flight and residence permit in current country.

Permissions to use the validated survey instruments were obtained from all copyright holders. The questionnaire, including the HSCL-10 and HTQ instruments and the questions from the HUNT and the HUBRO study, was forward translated from English to Standard Modern Arabic by two double-blinded professional translators before synthetizing and back-translation [26]. Challenging words and phrases were discussed within the translation team and with bilingual health workers. Finally, the questionnaire was piloted among a group of six Syrian refugees in a Norwegian asylum centre.

### Analytic approach

We calculated prevalence proportions with 95% confidence intervals (CI). Prevalence proportions were standardised to the age and gender composition of the Syrian population in Norway by the end of 2017 [6] in order to increase transferability of results. Data collected in both Lebanon and Norway was standardised in the same manner as the participants in Lebanon were resettlement refugees awaiting transfer for Norway. Sociodemographic and migration related exposures are presented as crude prevalence proportions, while all outcomes are presented as standardised prevalence proportions. We ran analyses of both daily and weekly use of drugs, but the latter did not differ substantially from the former and is not displayed here. We performed linear and logistic regression to study the relationship between factors related to forced migration and two outcomes: mental health problems and chronic pain. The explanatory variables time in transit, migrating alone, detainment during flight, lack of residence permit and trauma exposure were chosen to address different aspects of the experience of forced migration. All models are presented both crude and with adjustment for age, gender and country (Norway or Lebanon). The analyses were conducted using STATA/IC software, version 15.1, (StataCorp LLC, Texas, USA).

## Results

Overall, the study population had a mean age of 33 years (SD 10) with balanced gender distribution in Lebanon (51% women) and a predominance of men in Norway (27% women) (Table 1). The majority were Arabic speakers, with a 12% Kurmanji speaking minority. Respondents had a mean of 9 years (SD 4) of education. The participants had arrived in their current country of residence a mean of 4 years ago (SD 2). One in four reported to have passed a transit country and more than one quarter had migrated alone, although these figures diverged depending on the site. The crude prevalence of reported trauma exposure was 41% (37–44). Other sociodemographic and migration related characteristics are described in detail in Table 1.

**Table 1:** Sociodemographic, migration process and trauma related factors

	All	Lebanon	Norway
	***N*** **= 827**	***N*** **= 506**	***N*** **= 321**
SOCIODEMOGRAPHIC FACTORS			
Gender (women), n (%)	340 (41)	255 (51)	85 (27)
Age (years), mean (SD)	33 (10)	34 (9)	31 (11)
Mother tongue, n (%)			
Arabic	721 (87)	481 (95)	240 (75)
Kurmanji	99 (12)	22 (4)	77 (24)
Marital status, n (%)			
Married	533 (65)	377 (75)	156 (49)
Co-habiting with partner	524 (95)	372 (98)	152 (89)
Number of children, mean (SD)	2 (2)	3 (2)	1 (2)
Education (years), mean (SD)	9 (4)	8 (3)	11 (4)
MIGRATION AND TRAUMA RELATED FACTORS			
Time since flight (years), mean (SD)	4 (2)	5 (1)	3 (2)
Time since arrival (years), mean (SD)	4 (2)	5 (1)	2 (1)
Been in transit country, n (%)	215 (26)	30 (6)	185 (59)
Time in transit countries, n (%)			
Up to 6 months	69 (35)	4 (15)	65 (38)
6–12 months	27 (14)	3 (11)	24 (14)
1–2 years	32 (16)	4 (15)	28 (17)
> 2 years	68 (35)	16 (59)	52 (31)
Migrating alone, n (%)	231 (28)	75 (15)	156 (49)
Retained during flight, n (%)	58 (10)	1 (0)	57 (19)
Exposed to potentially traumatic event(s), n (%)	322 (41)	201 (41)	121 (40)
No residence permit in current country, n (%)	373 (46)	337 (67)	36 (11)

### Health status and use of medication

**Fig. 1:**
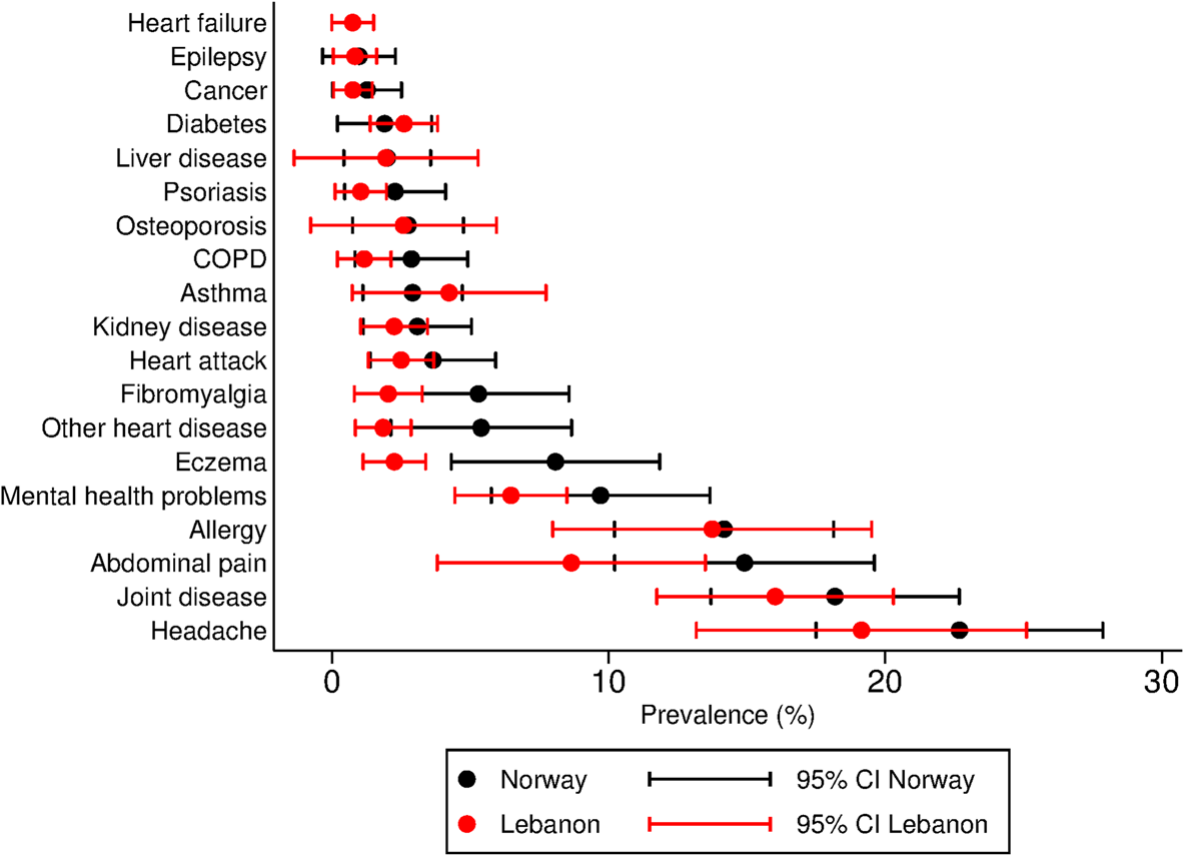
Lifetime prevalence proportions of selected conditions and symptoms with confidence intervals. Prevalence proportions standardised to the age and gender distribution of the total Syrian population in Norway by the end of 2017 [6]

About half of the participants reported that they had never had any of the health problems listed in the questionnaire. In both sites, headache was the most prevalent condition reported, followed by joint disorders, allergy, abdominal pain and mental health problems (Fig. 1, additional file 1). The standardised prevalence proportion of NCDs was 12% (10–15). Syrian women more frequently reported musculoskeletal complaints including arthritis, fibromyalgia and other joint disease (22% (17–28) and 32% (22–44) in Lebanon and Norway) compared to Syrian men (15% (11–20) and 17% (12–23) in Lebanon and Norway). There was only one case of tuberculosis and no reports of stroke or brain haemorrhage.

Overall, 30% (27–33) reported chronic pain (Table 2), while the percentages of chronic pain were 46% (38–54) among those reporting headaches, and 57% (48–64) among those reporting musculoskeletal pain. The prevalence of long-term illness or injury, lasting for more than one year and affecting daily life was 29% (26–33). The items measuring mental health showed satisfying internal consistency (Cronbach’s alfa for HSCL-10: 0.89 and for HTQ: 0.92). Symptoms indicating anxiety and/or depression were presented by 35% (32–38) of respondents (mean HSCL-10 score 1.62), while symptoms compatible with PTSD were revealed by 7% (5–9) (mean HTQ score 1.56).

**Table 2:** Prevalence of chronic pain, chronic impairment and mental health problems

	n	All^a^		Lebanon^a^		Norway^a^	
Chronic pain (Pain > 6 months) (%, CI)	772	30	(27–33)	31	(27–35)	37	(31–43)
Chronic impairment (> 1 year) (%, CI)	775	29	(26–33)	30	(26–34)	33	(27–38)
Anxiety/depr. (HSCL-10 cut-off 1.85) (%, CI)	788	35	(32–38)	31	(27–35)	33	(27–38)
HSCL-10 score (mean, CI)		1.62	(1.57–1.67)	1.56	(1.48–1.63)	1.62	(1.53–1.71)
PTSD diagnosis (HTQ cut-off 2.5) (%, CI)	696	7	(5–9)	4	(2–6)	7	(5–11)
HTQ score (mean, CI)		1.56	(1.52–1.60)	1.49	(1.44–1.55)	1.53	(1.46–1.60)

^a^Prevalence proportions and means with confidence intervals (CI) weighted by age and gender

**Fig. 2:**
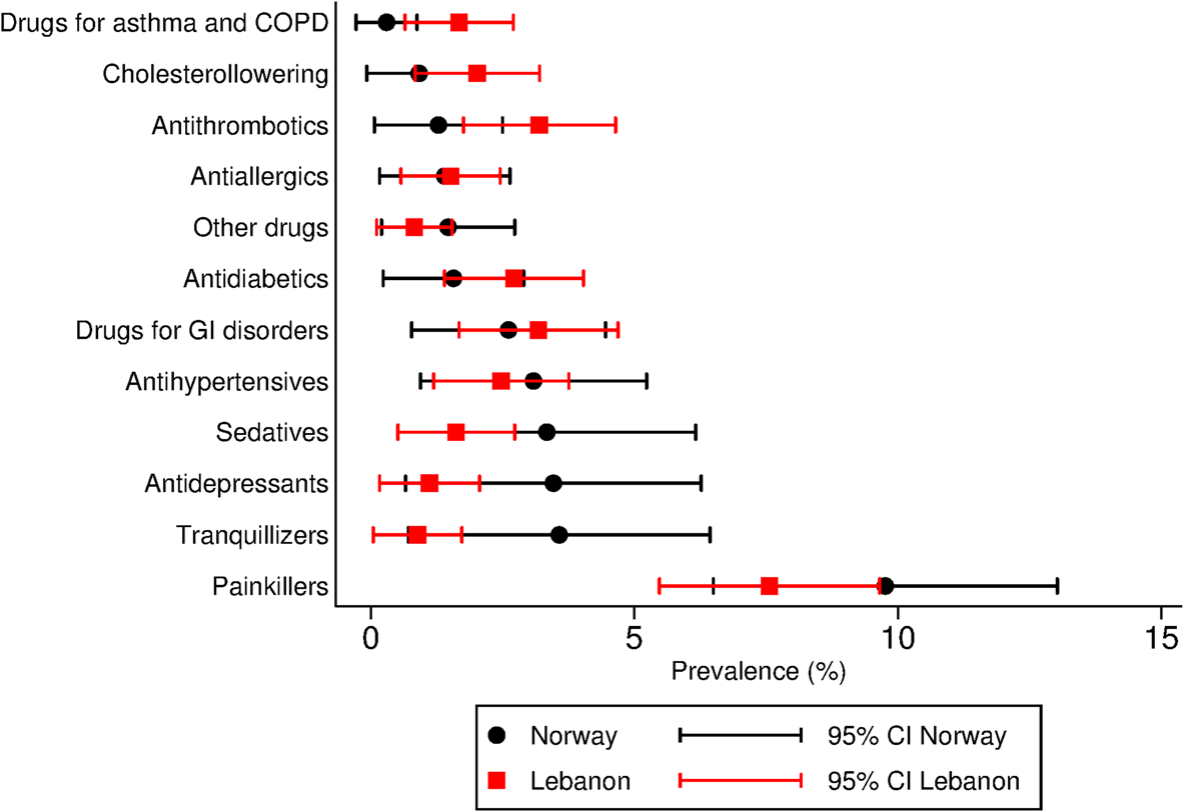
Prevalence proportions of daily use of medication use with confidence intervals. Prevalence proportions standardized to the age and gender distribution of the total Syrian population in Norway by the end of 2017 [6]

The drugs most frequently used on a daily base were painkillers (Fig. 2, additional file 2). Women used painkillers daily more readily than men. Among women 14% (10–19) in Lebanon and 13% (6–22) in Norway used painkillers daily, while corresponding rates among men were 4% (2–7) and 9% (5–14). Psychotropic drugs (antidepressants, sedatives and tranquillizers combined) were more commonly used in Norway and the difference was most profound among males.

Among those reporting cardiovascular disease, only 14% (6–27) used antihypertensives, 12% (4–25) used antithrombotic medication and 6% (1–18) used cholesterol lowering medication. The highest rate of medication usage was found among patients reporting diabetes mellitus, of which 42% (22–64) used antidiabetic drugs daily. Among those reporting headaches, 21% (15–28) reported using painkillers every day. Among those reporting mental health problems 17% (9–28) used one or more psychotropic drug.

### Association of migration related factors and health

Table 3 shows the association between factors related to forced migration and two prevalent outcomes among our respondents, mental health problems and chronic pain, using linear and logistic regression respectively (Table 3). Exposures to potentially traumatic events were associated with both symptoms of anxiety/depression and PTSD. Migrating alone was also associated with mental health problems both in crude and adjusted models. Detainment during flight did not show significant associations with anxiety/depression or PTSD symptoms neither before nor after adjustment, and lack of residence permit was associated with PTSD in the adjusted model only. Traumatic experiences were associated with chronic pain both in the unadjusted and adjusted model, but we found no statistically significant association between other migration related variables and reporting chronic pain.

**Table 3:** Associations between migration related factors and anxiety/depression symptoms, PTSD symptoms and and chronic pain

	Anxiety/depression symptoms		(PTSD symptoms)		Chronic pain	
	(Linear regression)		(Linear regression)		(Logistic regression)	
Variable	Crude β (95% CI)	Adj. β (95% CI) *	Crude β (95% CI)	Adj. β (95% CI) *	Crude OR (95% CI)	Adj. OR (95% CI) **
Gender (men ref.)	0.11 (0.02;0.20)	0.12 (0.02;0.21)	0.05 (−0.03;0.13)	0.06 (−0.02;0.14)	1.07 (0.79;1.45)	1.17 (0.84;1.61)
Age	0.00 (0.00;0.01)	0.00 (0.00;0.01)	0.01 (0.00–0.01)	0.01 (0.00;0.01)	1.03 (1.02;1.05)	1.04 (1.02;1.05)
Education	0.02 (0.01;0.03)	0.02 (0.01;0.03)	0.02 (0.01;0.02)	0.02 (0.01;0.03)	1.02 (0.99;1.06)	1.02 (0.98;1.06)
Country (Lebanon ref.)	0.00 (−0.09;0.10)	0.04 (− 0.06;0.14)	0.01 (− 0.07;0.09)	0.03 (− 0.05;0.12)	1.37 (1.01;1.86)	1.68 (1.20–2.34)
Time in transit	− 0.04 (− 0.07; − 0.01)	−0.05 (− 0.08;0.01)	−0.02 (− 0.05;0.01)	−0.04 (− 0.08; − 0.01)	1.09 (0.98;1.20)	1.03 (0.91;1.16)
Migrating alone	0.17 (0.07;0.27)	0.24 (0.13;0.36)	0.15 (0.06;0.24)	0.19 (0.09;0.29)	1.06 (0.76;1.48)	0.97 (0.66;1.42)
Detained during flight	0.18 (−0.00;0.36)	0.18 (−0.02;0.38)	0.05 (− 0.11;0.21)	0.08 (− 0.10;0.25)	1.48 (0.84;2.62)	1.71 (0.90;3.23)
No residence permit	0.03 (−0.06;0.12)	0.08 (−0.03;0.18)	0.06 (− 0.02;0.14)	0.11 (0.02;0.21)	0.76 (0.56;1.03)	0.90 (0.62;1.31)
Traumatic experiences	0.42 (0.34;0.51)	0.42 (0.33;0.51)	0.46 (0.38;0.54)	0.44 (0.37;0.52)	2.58 (1.88;3.52)	2.48 (1.79;3.43)

*β-coefficients (β) adjusted by age, gender and country. β values with confidence intervals above 0 indicate significant association

**Odds ratios (OR) adjusted by age, gender and country. OR with confidence interval values above 1 indicate significant association

## Discussion

Our study gives a unique overview of the health status and use of medication among Syrian refugees in two different migration phases: in a transit setting and in a recipient country. The prevalence proportion of NCDs in our sample is low. Yet a disproportionate number of those reporting NCDs does not seem to be under adequate treatment. The levels of psychological distress displayed in this study are similar to those reported among Syrian refugees elsewhere [27] while we lack comparators to the number reporting chronic pain. Further, our study confirms that migration related risk factors like exposure to traumatic events and migrating alone are associated with higher prevalence of mental health problems. As far as we know, a novel finding is the association between trauma exposure and chronic pain in an unselected population of refugees.

To our knowledge no other studies report *lifetime prevalence of* ill health among asylum seekers and refugees, nor among the general Syrian population. Studies of *point prevalence* among asylum seekers and refugees commonly report headache, injuries, and musculoskeletal, dermatological, dental, respiratory, gastrointestinal, and mental health problems to be frequent problems [13, 28–31]. Most of these studies evaluate clinic-based populations and rely on retrospective assessments of clinical records, making it impossible to extrapolate prevalence proportion to the population level. However, complaints of pain, particularly headache and musculoskeletal pain, seem to be a commonality.

In our material, approximately one third reported chronic pain lasting for more than 6 months. Further, almost one in three of our respondents reported chronic impairments lasting more than 1 year and impeding daily life. Comparing our data with the literature is challenging, as studies reporting chronic pain among refugees generally are restricted to selected groups such as torture survivors [32, 33]. Nevertheless, the high prevalence we find at the population level suggests that chronic pain and related impairment among forcibly displaced may be a barrier to integration that should be systematically addressed.

The prevalence proportions of NCDs in our sample are low compared to other studies among refugees from Syria. A study from Jordan found that 30% of the Syrian refugees surveyed had chronic disease [27], although the term “chronic disease” was not explicitly defined in the article. Another study reported that 22% of non-camp Syrian refugees in Jordan suffered from at least one NCD [13]. Concerning communicable disease, the incidence rate of tuberculosis in Syria has been estimated to 19 per 100,000 population [34]. In line with the relatively modest incidence rates in our respondent’s country of origin, there was only one case of reported tuberculosis in our sample. This finding supports the view that infectious diseases are not a major health problem among refugees from Syria. Indeed, a narrow focus on communicable diseases in health assessments of newly arrived refugees from this region may divert attention from more important health challenges.

The reported levels of psychological distress indicating anxiety and/or depression in this study correspond to levels reported among refugees in various sites (ranging from 32 to 44%) [35, 36], while the figure for PTSD symptoms is in the lower end of the range compared to prevalence proportions reported in systematic reviews and meta-analyses (ranging from 9 to 36%) [8, 37]. In a review looking particularly at Syrians in neighbouring countries, levels of PTSD ranged from 16 to 83% [38]. We do not have any full explanation for this comparatively low prevalence of PTSD but many of our respondents escaped the atrocities of the Syrian war at an early stage. Some papers report higher rates of depression and anxiety among asylum seekers compared to refugees, highlighting the impact of legal status, though in our material we only found evidence for such association between PTSD and lack of residence permit in the adjusted regression model. The relatively low level of PTSD symptoms in our sample contradicts the substantial share of our respondents exposed to potential traumatic events. However, factors acting protective to enhance resilience among refugees is not well understood. Further research is needed to identify interventions both outside and within the health care system that promote resilience, health and well-being among refugees exposed to traumatic events.

Regular use of medication may be challenging in the context of displacement. Previous studies from Jordan have shown that approximately a quarter of Syrian refugees in need of medication are lacking access to drugs [27] and interruptions in regular medication are predominantly due to restricted financial resources [13]. In our study, the access gap seems to be even broader. Health care providers involved in health assessments of refugees in transit and recipient settings should seek to identify discontinuity in use of regular medication and provide access to necessary drugs.

Migrating alone is a risk factor for adverse mental health outcomes in our study. Family separation has previously been shown to increase migration related stress [39]. Additionally, our study finds that exposure to potentially traumatic events is a risk factor not only for anxiety/depression and PTSD symptoms but also for chronic pain. The relationship between traumatic experiences and mental health problems is well established [40], and physical complaints are common among PTSD patients [41]. However, the association between trauma exposure and chronic pain in unselected refugee populations is poorly examined and should be further studied. We recommend that public health policies and practises address migration related risk factors among forcibly displaced individuals.

### Strengths and limitations

Our study gives a unique approximation to a population-based overview of the health status and use of medication among Syrian refugees in two different migration phases.

Our sampling protocol reflects the inherent challenges in studying moving populations. Although we consider the study a close proxy to population-based study, as opposed to studies recruiting patients from health clinics, we lack a complete sampling frame, as there is no central register of Syrian refugees on the move. The connection to educational activities for refugees may have affected the representativeness of our study population. Nevertheless, the educational activities were compulsory for the refugees at the time of recruitment. The high response rate increases the representativeness of the sample.

The demographic patterns at the two recruitment sites were, as expected, clearly divergent; the sample from Lebanon corresponds well with the Norwegian authorities’ official resettlement policy that gives explicit priority to families [42]. In parallel, the sample from Norway includes many who arrived as asylum seekers among which men are known to be overrepresented. We have therefore reported prevalence proportions standardised to the demographic patterns of the overall population of Syrians in Norway and we adjust for age, gender and country in our regression analyses. There are well-known gender differences in the prevalence of many of the conditions we explore, and we have demonstrated variance in the prevalence of musculoskeletal complaints and use of painkillers. For other conditions and drugs, we have only displayed results stratified by gender in the supplementary material as we find few gender differences. The sample size might be insufficient to detect minor differences in the prevalence proportions between women and men.

Importantly, this study presents findings of self-assessed health. To increase rigor, we employed predominantly validated survey items and validated translation and adaption principles.

## Conclusions

Forcibly displaced Syrians in Lebanon and Norway in general have few chronic diseases yet bear a high burden of chronic pain and symptoms of mental health problems. Moreover, disproportionate numbers of those reporting non-communicable disease do not seem to be under adequate treatment. Public health policymakers and practitioners need to ensure that universal health coverage also encompasses forcibly displaced individuals along their migration trajectories. Further investigations are warranted into the relationship between trauma exposure and chronic pain among forcibly displaced migrants as well as on culturally accepted and cost-effective interventions for central health challenges including chronic pain and trauma related psychological morbidity.

## Supplementary information

**Additional file 1.** Title of data: Lifetime prevalence of different health conditions (%). Description of data: table with lifetime prevalence proportions of selected health conditions and symptoms combined and stratified by country and gender.

**Additional file 2.** Title of data: Daily use of drugs (%). Description of data: table with prevalence proportions of daily use of selected drugs combined and stratified by country and gender.

## Data Availability

The datasets generated during the current study are not publicly available due to data protection regulations in Norway.

## Abbreviations

HSCL-10: Hopkins Symptom Checklist 10
HTQ: Harvard Trauma Questionnaire
HUBRO: Oslo Health Study
HUNT: Nord-Trøndelag Health Study
NCD: Non-communicable disease
UNHCR: United Nations High Commissioner for Refugees
