# Supplementary material for: Health status and use of medication and their association with migration related exposures among Syrian refugees in Lebanon and Norway: a cross-sectional study
Source: BMC Public Health. 2020 Mar 17;20:341. doi: 10.1186/s12889-020-8376-7 (PMC7077130; doi:10.1186/s12889-020-8376-7)
Supplement: Supplementary file 1 — Additional file 1. Title of data: Lifetime prevalence of different health conditions (%). Description of data: table with lifetime prevalence proportions of selected health conditions and symptoms combined and stratified by country and gender. [file 12889_2020_8376_MOESM1_ESM.docx]

Additional file 1 Lifetime prevalence of different health conditions (%)

|  |  | All* | | Lebanon* | | Norway* | |
| --- | --- | --- | --- | --- | --- | --- | --- |
| Condition | **n** | **Prevalence (%)** | **95% CI** | **Prevalence (%)** | **95% CI** | **Prevalence (%)** | **95% CI** |
| Headache | 772 | 19 | (16-22) | 19 | (15-23) | 22 | (17-27) |
| Joint disease | 773 | 16 | (13-19) | 16 | (13-20) | 18 | (13-23) |
| Allergy | 776 | 13 | (11-16) | 14 | (11-17) | 12 | (9-17) |
| Abdominal pain/ diarrhea | 769 | 11 | (9-13) | 9 | (6-12) | 16 | (12-21) |
| Mental ill health | 770 | 9 | (7-11) | 6 | (4-9) | 10 | (7-14) |
| Eczema | 777 | 5 | (3-6) | 2 | (1-4) | 8 | (5-12) |
| Asthma | 778 | 4 | (3-6) | 4 | (3-6) | 3 | (1-6) |
| Heart attack or chest pain | 776 | 3 | (2-5) | 2 | (1-4) | 4 | (2-7) |
| Kidney disease | 777 | 3 | (2-4) | 2 | (1-4) | 3 | (1-6) |
| Fibromyalgia | 774 | 3 | (2-4) | 2 | (1-4) | 6 | (3-9) |
| Other heart disease | 777 | 3 | (2-4) | 2 | (1-3) | 6 | (3-9) |
| Diabetes | 781 | 2 | (1-4) | 3 | (1-5) | 2 | (1-4) |
| COPD | 778 | 2 | (1-3) | 1 | (0-3) | 2 | (1-4) |
| Psoriasis | 778 | 1 | (1-3) | 1 | (0-2) | 2 | (1-5) |
| Osteoporosis | 777 | 1 | (1-2) | 1 | (0-2) | 2 | (1-5) |
| Liver disease | 777 | 1 | (1-2) | 2 | (1-4) | 2 | (1-5) |
| Cancer | 778 | 1 | (0-2) | 1 | (0-2) | 1 | (0-4) |
| Epilepsy | 773 | 1 | (0-2) | 1 | (0-2) | 1 | (0-3) |
| Heart failure | 777 | 0 | (0-1) | 1 | (0-2) | 0 | - |
| Stroke | 778 | 0 | - | 0 | - | 0 | - |
| Tuberculosis | 779 | 0 | - | 0 | - | 0 | - |
|  |  |  |  |  |  |  |  |
|  | **All women**** | | **Women in Lebanon**** | | **Women in Norway**** | |  |
| Condition | **Prevalence (%)** | **95% CI** | **Prevalence (%)** | **95% CI** | **Prevalence (%)** | **95% CI** |  |
| Headache | 26 | (21-31) | 23 | (18-29) | 27 | (17-39) |  |
| Joint disease | 22 | (17-27) | 22 | (17-28) | 27 | (17-38) |  |
| Allergy | 13 | (9-17) | 14 | (10-19) | 11 | (5-21) |  |
| Abdominal pain/ diarrhea | 10 | (7-14) | 8 | (5-12) | 13 | 6-23) |  |
| Mental ill health | 9 | (6-13) | 10 | (6-14) | 5 | (1-13) |  |
| Eczema | 6 | (4-10) | 6 | (3-10) | 11 | (5-20) |  |
| Asthma | 3 | (1-6) | 3 | (1-7) | 1 | (0-7) |  |
| Heart attack or chest pain | 3 | (1-5) | 3 | (1-6) | 3 | (0-11) |  |
| Kidney disease | 3 | (2-6) | 3 | (1-6) | 4 | (1-12) |  |
| Fibromyalgia | 3 | (1-6) | 2 | (1-5) | 5 | (1-13) |  |
| Other heart disease | 3 | (1-6) | 3 | (1-6) | 3 | (1-11) |  |
| Diabetes | 3 | (2-6) | 4 | (2-8) | 1 | (0-7) |  |
| COPD | 1 | (0-2) | 1 | (0-3) | 0 | . |  |
| Psoriasis | 1 | (1-3) | 1 | (0-3) | 4 | (1-12) |  |
| Osteoporosis | 2 | (1-5) | 2 | (0-4) | 4 | (1-12) |  |
| Liver disease | 0 | (0-2) | 1 | (0-3) | 0 | . |  |
| Cancer | 1 | (0-4) | 1 | (0-3) | 2 | (0-9) |  |
| Epilepsy | 1 | (0-2) | 0 | (0-2) | 2 | (0-9) |  |
| Heart failure | 0 | (0-2) | 0 | (0-2) | 0 | . |  |
| Stroke | 0 | - | 0 | . | 0 | . |  |
| Tuberculosis | 0 | - | 0 | . | 0 | . |  |
|  |  |  |  |  |  |  |  |
|  | **All men**** | | **Men in Lebanon**** | | **Men in Norway**** | |  |
| Condition | **Prevalence (%)** | **95% CI** | **Prevalence (%)** | **95% CI** | **Prevalence (%)** | **95% CI** |  |
| Headache | 16 | (13-20) | 17 | (12-23) | 19 | (14-25) |  |
| Joint disease | 13 | (10-17) | 13 | (9-18) | 13 | (9-19) |  |
| Allergy | 13 | (10-17) | 14 | (9-19) | 13 | (9-19) |  |
| Abdominal pain/ diarrhea | 11 | (8-15) | 9 | (6-14) | 17 | (12-23) |  |
| Mental ill health | 9 | (6-12) | 5 | (2-8) | 12 | (8-18) |  |
| Eczema | 4 | (2-6) | 1 | (0-3) | 7 | (4-12) |  |
| Asthma | 4 | (3-7) | 5 | (2-8) | 4 | (2-8) |  |
| Heart attack or chest pain | 3 | (2-6) | 2 | (1-5) | 4 | (2-7) |  |
| Kidney disease | 3 | (1-5) | 2 | (0-4) | 3 | (1-6) |  |
| Fibromyalgia | 3 | (1-5) | 2 | (1-5) | 6 | (3-10) |  |
| Other heart disease | 2 | (1-4) | 1 | (0-3) | 7 | (4-12) |  |
| Diabetes | 2 | (1-4) | 2 | (1-5) | 2 | (0-5) |  |
| COPD | 2 | (1-4) | 1 | (0-4) | 2 | (1-6) |  |
| Psoriasis | 1 | (1-3) | 1 | (0-3) | 2 | (0-4) |  |
| Osteoporosis | 1 | (0-2) | 0 | (0-2) | 1 | (0-4) |  |
| Liver disease | 2 | (1-4) | 3 | (1-6) | 3 | (1-7) |  |
| Cancer | 1 | (0-2) | 1 | (0-3) | 1 | (0-4) |  |
| Epilepsy | 1 | (0-2) | 1 | (0-3) | 1 | (0-3) |  |
| Heart failure | 1 | (0-2) | 1 | (0-3) | 0 | . |  |
| Stroke | 0 | - | 0 | . | 0 | . |  |
| Tuberculosis | 0 | - | 0 | . | 0 | . |  |

*Prevalence and confidence intervals (CI) weighted by age and gender

** Prevalence and confidence intervals (CI) weighted by age
