# Supplementary material for: Health status and use of medication and their association with migration related exposures among Syrian refugees in Lebanon and Norway: a cross-sectional study
Source: BMC Public Health. 2020 Mar 17;20:341. doi: 10.1186/s12889-020-8376-7 (PMC7077130; doi:10.1186/s12889-020-8376-7)
Supplement: Supplementary file 2 — Additional file 2. Title of data: Daily use of drugs (%). Description of data: table with prevalence proportions of daily use of selected drugs combined and stratified by country and gender. [file 12889_2020_8376_MOESM2_ESM.docx]

Additional file 2 Daily use of drugs (%)

|  |  | All* | | Lebanon* | | Norway* | |
| --- | --- | --- | --- | --- | --- | --- | --- |
| Drug | **n** | **Prevalence (%)** | **95% CI** | **Prevalence (%)** | **95% CI** | **Prevalence (%)** | **95% CI** |
| Painkillers | 776 | 9 | (7-11) | 7 | (5-10) | 10 | (7-14) |
| Drugs for gastrointestinal conditions | 765 | 3 | (2-5) | 3 | (2-5) | 3 | (1-5) |
| Antithrombotics | 768 | 3 | (2-4) | 3 | (2-5) | 1 | (0-3) |
| Antihypertensives | 770 | 3 | (2-4) | 2 | (1-4) | 3 | (1-6) |
| Antidiabetics | 770 | 2 | (1-4) | 3 | (1-5) | 2 | (1-4) |
| Sedatives | 768 | 2 | (1-3) | 2 | (1-3) | 3 | (2-6) |
| Antiallergics | 764 | 2 | (1-3) | 2 | (1-3) | 1 | (0-3) |
| Antidepressants | 767 | 2 | (1-3) | 1 | (0-3) | 4 | (2-6) |
| Cholesterol lowering | 769 | 2 | (1-3) | 2 | (1-4) | 1 | (0-3) |
| Tranquillizers | 769 | 2 | (1-3) | 1 | (0-2) | 4 | (2-7) |
| Other drugs | 768 | 1 | (1-2) | 1 | (0-2) | 1 | (0-4) |
| Drugs for asthma and COPD | 770 | 1 | (1-2) | 1 | (1-3) | 0 | (0-2) |
|  |  |  |  |  |  |  |  |
|  | **All women**** | | **Women in Lebanon**** | | **Women in Norway**** | |  |
| Drug | **Prevalence (%)** | **95% CI** | **Prevalence (%)** | **95% CI** | **Prevalence (%)** | **95% CI** |  |
| Painkillers | 14 | (10-18) | 14 | (10-19) | 13 | (6-22) |  |
| Drugs for gastrointestinal conditions | 5 | (3-8) | 5 | (3-9) | 5 | (1-12) |  |
| Antithrombotics | 2 | (1-5) | 3 | (1-6) | 0 | . |  |
| Antihypertensives | 2 | (1-5) | 1 | (0-4) | 5 | (1-12) |  |
| Antidiabetics | 2 | (1-5) | 3 | (1-6) | 1 | (0-6) |  |
| Sedatives | 2 | (1-4) | 3 | (1-6) | 2 | (0-7) |  |
| Antiallergics | 2 | (1-4) | 3 | (1-6) | 0 | . |  |
| Antidepressants | 2 | (1-4) | 1 | (0-4) | 3 | (0-9) |  |
| Cholesterol lowering | 2 | (1-5) | 2 | (1-5) | 1 | (0-7) |  |
| Tranquillizers | 1 | (0-3) | 1 | (0-3) | 2 | (0-7) |  |
| Other drugs | 2 | (1-5) | 2 | (1-4) | 3 | (0-9) |  |
| Drugs for asthma and COPD | 2 | (1-4) | 2 | (1-5) | 0 | . |  |
|  |  |  |  |  |  |  |  |
|  | **All men**** | | **Men in Lebanon**** | | **Men in Norway**** | |  |
| Drug | **Prevalence (%)** | **95% CI** | **Prevalence (%)** | **95% CI** | **Prevalence (%)** | **95% CI** |  |
| Painkillers | 7 | (5-10) | 4 | (2-7) | 9 | (5-14) |  |
| Drugs for gastrointestinal conditions | 2 | (1-4) | 2 | (1-5) | 2 | (0-5) |  |
| Antithrombotics | 3 | (1-5) | 3 | (1-6) | 2 | (1-5) |  |
| Antihypertensives | 3 | (1-5) | 3 | (1-6) | 2 | (1-6) |  |
| Antidiabetics | 2 | (1-4) | 3 | (1-6) | 2 | (1-5) |  |
| Sedatives | 2 | (1-4) | 1 | (0-4) | 4 | (2-8) |  |
| Antiallergics | 2 | (1-4) | 1 | (0-3) | 2 | (0-5) |  |
| Antidepressants | 2 | (1-4) | 1 | (0-3) | 4 | (2-8) |  |
| Cholesterol lowering | 2 | (1-3) | 2 | (1-5) | 1 | (0-3) |  |
| Tranquillizers | 2 | (1-4) | 1 | (0-3) | 5 | (2-8) |  |
| Other drugs | 1 | (0-2) | 0 | (0-2) | 1 | (0-3) |  |
| Drugs for asthma and COPD | 1 | (0-2) | 1 | (0-3) | 0 | (0-3) |  |

*Weighted by age and gender

**Weighted by age
